# 

**Published:** 1887-07

**Authors:** 


### CONTENTS:

ARTICLE I.—The Types of Teeth in Disease.
By James F. Rymer, M. R. C. S. .................... 97

" II.—Antiseptics and Disinfectants in Office Practice.
By Alfred T. Peete.............. .......................... 104

" III.—Lectures on Dentistry for Popular Audiences ............. 109

" IV.—Pathology of Dental Caries. By E. Parsons, D. D. S.... 112

" V.—" Blind Abscess." By J. A. Thornton, D. D. S...... ..... 115

" VI.—Clamp Plate. By Dr. W. G. Browne....... ........... ..... 118

" VII.—Extracts from Lectures on Operative Dental Surgery.
By Wm. St. George Elliott, M. D., D. D. S ..... 120

" VIII.—Amalgam and Matrix. By Dr. Frank W. Low........... 126

" IX.—Observations and Experiments on the Action of certain Anæsthetics, Alone or Mixed, on the Fibres of the Fifth Pair of Nerves. By Drs. Lawrence Turnbull, E P. Reichert and John D. Thomas..................... 129

ARTICLE X.—Immediate Root-Filling.
By J. Smith Dodge, Jr. M. D., D. D. S ............ 134

" XI.—Women in Dentistry. By Mrs. R. B. Ramsey, D. D. S. 136

The American Dental Association. ...... ........... ............................. 139

Missouri State Dental Association.............................................. 140

### MONTHLY SUMMARY.

Proximal Decays.......... ................................................ .......... 141

A Method of Testing the Vitality of a Tooth's Pulp ....................... 141

Dr. Richardson on the Painless Extinction of Life in the Lower Animals. 142

Composition for Duplicating Models........................................... 143

Dentistry Recognized by the American Medical Association as a Specialty of Medicine.......................................................... 143

Gold Solder................................................................ .......... 144

Obtunding Sensibility................................................ .... 144

